# Colistin Sulfate Chiral Stationary Phase for the Enantioselective Separation of Pharmaceuticals Using Organic Polymer Monolithic Capillary Chromatography †

**DOI:** 10.3390/molecules24050833

**Published:** 2019-02-26

**Authors:** Ali Fouad, Montaser Sh. A. Shaykoon, Samy M. Ibrahim, Sobhy M. El-Adl, Ashraf Ghanem

**Affiliations:** 1 Chirality Program, Faculty of Science and Technology, University of Canberra, Canberra, ACT 2601, Australia; alifouad247@gmail.com; 2 Pharmaceutical Chemistry Department, Faculty of Pharmacy, Al-Azhar University, Assiut 71524, Egypt; monoceutical@yahoo.com; 3 Pharmaceutical Chemistry Department, Faculty of Pharmacy, Zagazig University, Zagazig 44519, Egypt; dr-samy2010@hotmail.com (S.M.I.); sobhyeladl@yahoo.com (S.M.E.-A.)

**Keywords:** colistin sulfate, enantioselective, encapsulation, capillary chromatography, monolith, organic polymer

## Abstract

A new functionalized polymer monolithic capillary with a macrocyclic antibiotic, namely colistin sulfate, as chiral selector was prepared via the copolymerization of binary monomer mixtures consisting of glycidyl methacrylate (GMA) and ethylene glycol dimethacrylate (EGDMA) in porogenic solvents namely 1-propanol and 1,4-butanediol, in the presence of azobisiso-butyronitrile (AIBN) as initiator and colistin sulfate. The prepared capillaries were investigated for the enantioselective nano-LC separation of a group of racemic pharmaceuticals, namely, α- and β-blockers, anti-inflammatory drugs, antifungal drugs, norepinephrine-dopamine reuptake inhibitors, catecholamines, sedative hypnotics, antihistaminics, anticancer drugs, and antiarrhythmic drugs. Acceptable separation was achieved for many drugs using reversed phase chromatographic conditions with no separation achieved under normal phase conditions. Colistin sulfate appears to be useful addition to the available macrocyclic antibiotic chiral phases used in liquid chromatography.

## 1. Introduction

Most of the drugs currently in use are worldwide marketed as racemates. Enantiomers can exhibit different activities in biological systems, in particular, their pharmacology, toxicology, pharmacokinetics and metabolism. Therefore, it is important to separate single enantiomers to limit side effects that may arise from unwanted enantiomers [1,2,3]. To access enantiomerically pure compounds, enantioselective chromatographic techniques have been considered as the most feasible method compared to other more expensive and time-consuming approaches [4,5,6,7,8,9,10]. Among these techniques, High Performance Liquid Chromatography (HPLC) is the most widely used technique in enantiomer separation [11,12,13]. In HPLC, a chiral selector is required to form a Chiral Stationary Phase (CSP), the main driver for the chiral separation. The CSP is normally bound, immobilized adsorbed or otherwise attached to an appropriate support. The enantiomers are then resolved by the formation of temporary diastereomeric complexes between the analyte and the CSP. The stationary phase support plays a very important role in any research investigation in this field [14,15,16].

Because of their advantages, the use of monoliths as stationary phases for HPLC represents a promising alternative to particle packed columns for CEC, conventional HPLC columns and nano-HPLC capillaries [17,18,19,20,21]. The preparation of organic polymer monolithic stationary phases via surface modification with a suitable precursor followed by the polymerization process results in increased stability of the monolith and affords greater adherence to the confining wall [22,23,24,25,26,27,28]. Many CSPs have been previously reported attached to monolithic support for chiral separation, especially in enantioselective capillary chromatography [29,30,31]. However, only a few were macrocyclic antibiotics [32].

Natural macrocyclic antibiotic materials play a very important role in chiral separation as useful CSPs. In general, the macrocyclic antibiotics most widely used as chiral selectors are vancomycin, vancomycin aglycon, norvancomycin, teicoplanin, and teicoplanin aglycon, ristocetin A, thiostrepton, rifamycin, kanamycin, streptomycin, fradiomycin, eremomycin and avoparcin [33]. The unique features of these chiral selectors include different chiral centers, inclusion cavities, phenyl rings, several hydrogen donor and acceptor sites, sugar moieties, and other groups which are the main drivers for their good chiral recognition abilities in different chromatographic modes. The chiral recognition mechanism in most of these antibiotics chiral selectors relies heavily on complexation, hydrogen bonding, inclusion complex formation, dipole interactions, steric interactions, and anionic and cationic binding. These chiral selectors have been employed for the enantiomeric resolution of a variety of racemates in HPLC, CEC, and CE [34,35,36,37]. Furthermore, a few were previously used in preparation of chiral monolithic columns for the enantioselective separation of racemic pharmaceuticals [38]. Colistin sulfate represents a new addition to the macrocyclic antibiotic family enabling its multi-chirality sites and functional groups to provide chromatographic interactions with racemic analytes [38]. Furthermore, the encapsulation of a macrocyclic antibiotic in an organic polymer monolith in capillary HPLC hasn’t been previously reported. The ease of in situ preparation in capillaries or narrow channels of microfluidic devices render these ideal stationary phases for microscale separation formats [39].

Here we introduce a new chiral macrocyclic antibiotic, namely colistin sulfate, encapsulated in organic polymer monolithic capillary for the enantioselective nano-liquid chromatographic separation of a set of racemic pharmaceuticals.

## 2. Results

### 2.1. Preparation and Characterization of Polymer Monoliths

**Figure 1:**
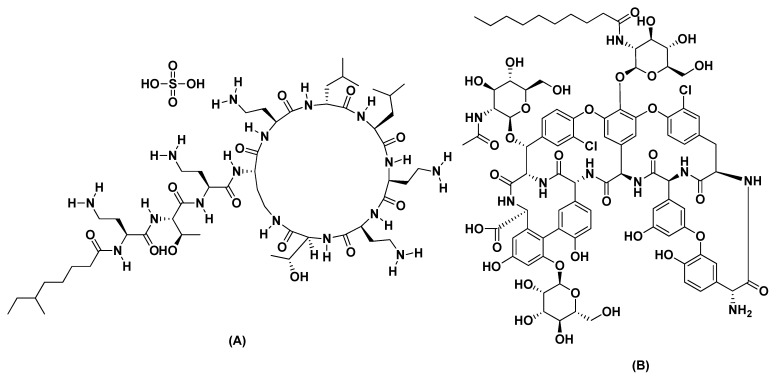
Chemical structure of colistin sulfate (**A**) and teicoplanin (**B**).

The use of macrocyclic antibiotics in chiral separation was previously reported in both conventional HPLC and CEC. In particular, macrocyclic antibiotic-based silica monolithic columns were previously studied [32,38,40]. However, the macrocyclic antibiotics were immobilized on the activated monoliths by a tedious reductive amination process [32]. No work was previously reported on the polymer monolithic antibiotic-based CSP in capillary liquid chromatography. Here we report the first use of colistin sulfate as a macrocyclic antibiotic chiral selector entrapped in organic polymer monolith for enantioselective capillary LC (Figure 1). The miscibility and solubility of colistin sulfate was tested in porogenic solvents used in monolith preparation, namely 1,4-butanediol, ethanol and *n*-propanol. When 1,4-butanediol was used in the polymerization mixture, a highly homogeneous solution occurred, however, better solubility was achieved when used in combination with 1-propanol as porogenic solvent.

Colistin sulfate-based polymer monolithic column (C1) was prepared via in situ copolymerization of colistin sulfate with monomers (40%) (GMA (20%) as a functional monomer and EGDMA (20%) as a cross linker) in the presence of a ternary porogenic system composed of 1-propanol (48%), 1,4-butanediol (6%) and chiral selector (6%). The ratio of the monomers to the porogens was fixed at 40:60 *w*/*w*, respectively; this was selected to provide columns with a good balance of permeability, surface area and mechanical stability.

#### Scanning Electron Microscopy (SEM) and Surface Properties of the Monoliths

**Figure 2:**
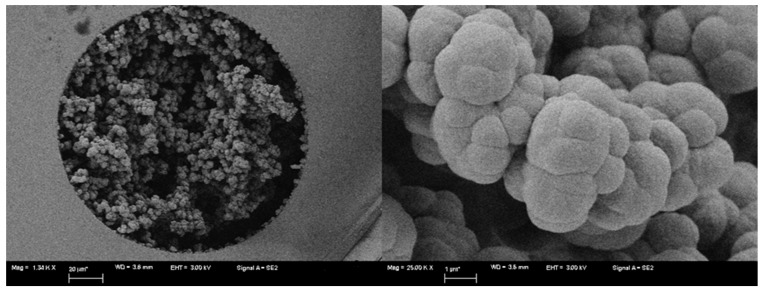
SEM of column C1 at 1200× and 25,000× (left and right, respectively) shows small micro-globules with rough surface.

Scanning electron microscopy (SEM) photos were taken to study the morphology of the prepared monolith. Column C1 showed a porous structure with interconnecting channels allowing the flow of mobile phase with reduced column back-pressure (Figure 2). The textural surface properties of the monolithic columns, including the specific surface area and the pore structure, were previously calculated by our group. The pore size distribution was determined from the adsorption isotherms using the Barrett–Joyner–Halenda (BJH) method. Specific surface area (SBET) was calculated using multi-point adsorption data from a linear segment of the N_2_ adsorption isotherms using the Brunauer–Emmett–Teller (BET) theory [41]. The monolithic column previously prepared using similar procedure and demonstrated good enantioseparation exhibited surface area of 28.67 m^2^/g, pore size of 169.2 nm and total pore volume of 0.12 cm^3^/g.

Elemental analysis was used to determine the nitrogen (1.9 and 2.68 % *w*/*w*) and sulfur (0 and 0.4% *w*/*w*) content in the C1 column and blank column (G column), respectively. The blank column (G column) was prepared using the same polymerization mixture with addition of water instead of colistin sulfate. The measured the nitrogen contents were 1.9 and 2.68 % *w*/*w* and the measured sulfur contents were 0 and 0.4% *w*/*w* in the C1 column and blank column (G column), respectively. Elemental analysis was conducted to ensure the relevance of the presence of colistin sulfate in the prepared C1 column. These results confirm the presence of the chiral selector in the prepared C1 column.

### 2.2. Enantioseparation of Different Classes of Pharmaceutical Racemates

**Figure 3:**
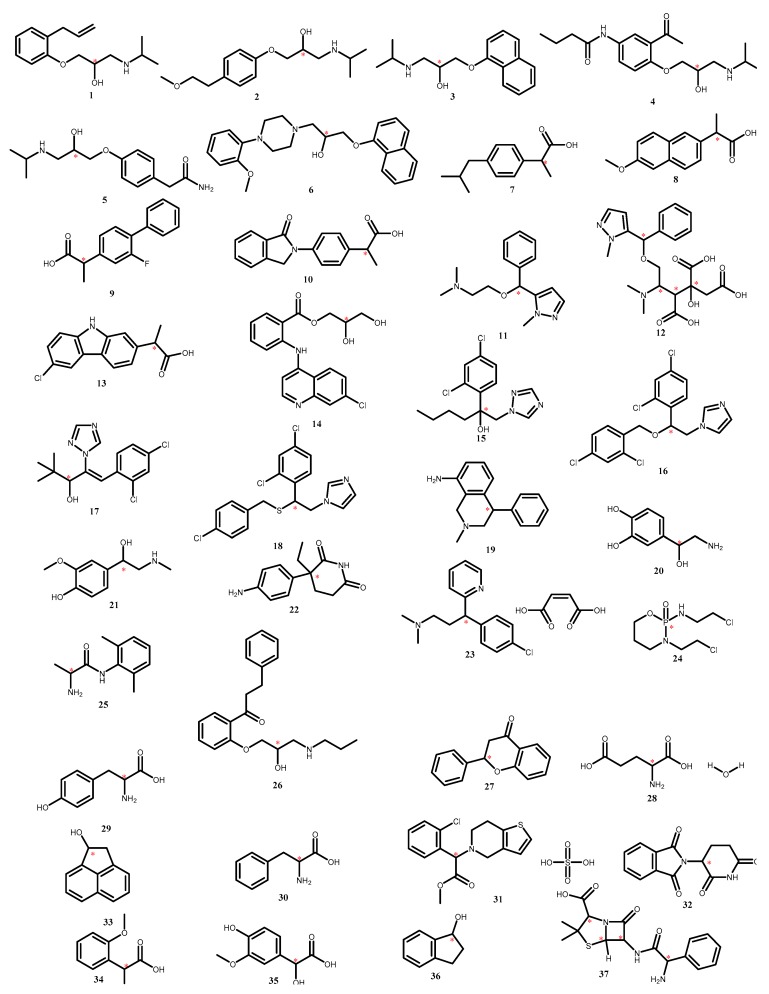
Chemical structures of the investigated racemates.

**Figure 4:**
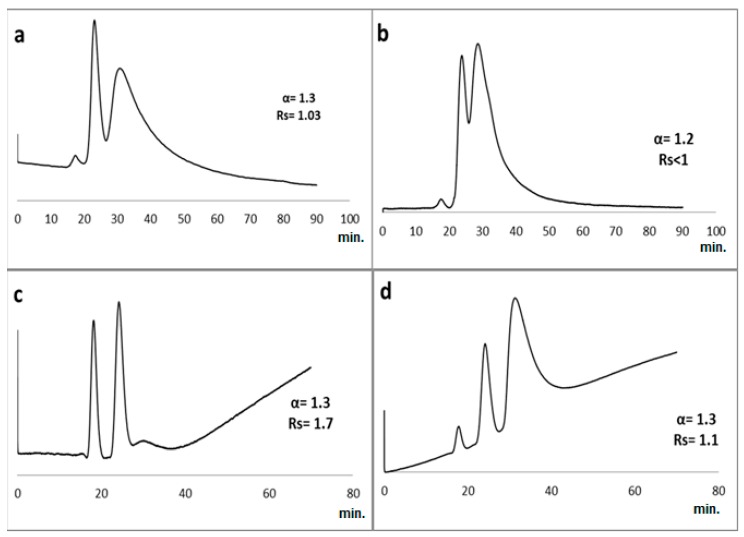
Enantioselective nano-LC separation of; (**a**) racemic 4-hydroxy-3-methoxymandelic acid (**35**); (**b**) phenylalanine (**30**) (mobile phase: methanol/water 40:60 *v*/*v*,); (**c**) tyrosine (**29**) and (**d**) *O*-methoxymandelic acid (**34**) on a C1 capillary column (150 µm ID, 25 cm length). UV: 219 nm, flow rate: 1 µL/min.

The colistin sulfate-based polymer monolithic capillary column was prepared as described above and investigated for the nano-LC enantioseparation of a set of different classes of racemic pharmaceuticals, namely: β-blockers, α-blockers, anti-inflammatory drugs, antifungal drugs, norepinephrine-dopamine reuptake inhibitors, catecholamines, sedative hypnotics, antihistamines, antibacterial drugs, anticancer drugs and antiarrhythmic drugs. Although reversed phase enantio-selective LC examples are limited, macrocyclic antibiotics were previously used in enantioseparation chromatography under reversed phase chromatographic mode [34,36,37,38,42,43,44,45]. The initial mobile phase selected for the enantioseparation separation of racemates **1**–**37** (Figure 3) was a binary mixture of methanol/water screened from 95:5 to 5:95 *v*/*v* at 1 mL/min flow rate at fixed UV detection 219 nm with eleven compounds separated (Rs ≥ 1) (Table 1). For examples, in MeOH/H_2_O 80:20 *v*/*v*, only ibuprofen (**7**) was separated, while in MeOH/H_2_O 40:60, indoprofen (**10**), hexaconazole (**15)** and miconazole (**16**) were separated. In MeOH/H_2_O 10:90 *v*/*v*, aminoglutethimide (**22),** tyrosine (**29**) and *O*-methoxymandelic acid (**34**) were also separated. The addition of an additive, namely triethylamine (TEA) 1% *v*/*v* in 10:90, resulted in the separation of acebutolol (**4**) normetanephrine (**21**), propafenone (**26**), tyrosine (**29**) and 4-hydroxy-3-methoxymandelic acid (**35**) (Figure 4), while non-acceptable separations were achieved by addition of the acidic additive namely trifluoroacetic acid (TFA). In an attempt to use normal phase namely *n*-hexane/2-propanol mixture ranging from 10–90% (*v*/*v*) resulted in resolution less than 1. All chromatographic data are summarized in Table 1.

Because of the novelty associated with using colistin sulfate as a chiral selector, confirmatory tests were done by injecting the separated enantiomeric drugs using capillary monolithic column without chiral selector (blank column, cf. Figure 1). The injected drugs included tyrosine (**29**), phenylalanine (**30**), *O*-methoxymandelic acid (**34**) and 4-hydroxy-3-methoxymandelic acid (**35**). Only single peaks were obtained under chromatographic conditions similar to those previously used when using capillary columns with colistin sulfate as CSP (C1 column). Furthermore, the single *S*-enantiomer of acebutolol (*S*-acebutolol) was injected on the C1 column (Figure 2). Only a single peak was obtained when used alone and mixed with its racemic mixture, which resulted in a peak with higher intensity, but unfortunately with low resolution. Also it was observed that *S*-acebutolol eluted first in the same retention time as the eluted single peak of single isomer *S*-acebutolol. The results achieved from the injection of the enantiomers on both the blank and C1 column, confirm the presence of the chiral selector in situ the capillary and that it was not washed out or dissolved in the mobile phase. The investigated repeatability of the used C1 column is considered as a proof of stability of the chiral selector contained in the capillary. It was also observed that the chiral separation was mostly achieved at high water content in the mobile phase; although, the chiral selector itself can be dissolved in water. This does not contradict what has been previously reported in literature where similar solvent used for dissolving the chiral selector can be used as mobile phase in the same column [32].

The combination of thin-hair capillary format in capillary HPLC is also beneficial as swapping from existing conventional liquid chromatography LC (mL flow, more solvent) to micro/nano flow LC (less solvent) is beneficial. The expected outcome will be environmentally responsible, cost effective and efficient analytical sample preparation and separation technologies for analytical laboratories throughout the world. Some featured benefits include but not limited to: (1) up to 4× increase in sensitivity; (2) improved turn-around-time with up to 5× faster separations; (3) up to a 95% reduction in mobile phase consumption and (4) improved robustness–less sample introduced into the MS when used in LC/MS and, ultimately, less instrument downtime.

For example, the chiral analysis for one run in conventional HPLC consumes at least 20–30 mL of environmentally unfriendly solvents for 30 min separation. On the other hand, in nano-HPLC, running a similar analysis under reversed phase conditions consumes less than 100 µL of water-based mobile phase. The capillary monolithic column is 10,000 less in internal diameter and operates with one million times less solvent volume than a conventional column. Consequently, this approach is economically efficient where only a single sorbent, namely colistin antibiotic, was used as CSP in capillary HPLC reducing materials/solvents consumption. Taking ibuprofen as an example, it was efficiently separated on the prepared monolithic column (Rs = 1.02) while it was recently enantio-separated (Rs = 1.05) on a mixed sorbents containing eremomycin and bovine serum albumin BSA-based CSP under reversed phase conditions using mobile phase: MeOH:KH_2_PO_4_ (0.1 M, pH 4.5); 50:50 (*v*/*v*); flow rate: 0.5 mL/min; and fixed UV 220 nm [34]. Another example is the recent use of mixed chiral sorbents based on silica with immobilized macrocyclic antibiotics eremomycin and vancomycin for the enantioselective of β-blockers such as atenolol and amino acids like phenylalanine by conventional HPLC using a mobile phase of MeOH:ACN–TEAA (0.1%, pH 4.5) (95:5, *v*/*v*), and a flow rate of 1 mL/min [37]. Nano-HPLC chromatograms for some of the separated compounds under different ratios of methanol and water are given in Figure 4.

### 2.3. Column Repeatability

**Figure 5:**
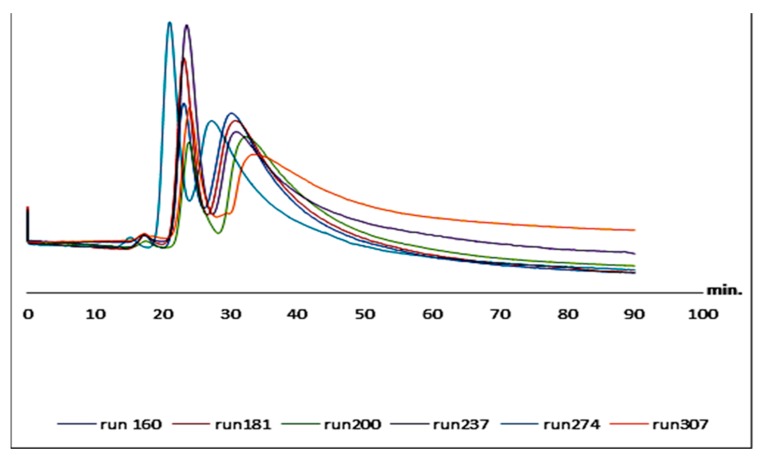
The loadability of the monolithic columns of 4-hydroxy-3-methoxymandelic acid (**35**) started at run no. 160 up to run no. 307, on C1 capillary column (150 µm ID, 25 cm length). mobile phase: methanol/water 40:60 *v*/*v*, UV: 219 nm, flow rate: 1 µL/min.

The repeatability is the ability to prepare equally performing capillaries at different time (run to run) and locations (batch to batch). To determine the repeatability of the prepared capillaries, two capillaries were prepared on the same day using the same polymerization mixture to test column-to-column repeatability. Moreover, batch-to-batch repeatability was tested by preparing three different batches at different days using the same polymer mixtures. 4-Hydroxy-3-methoxymandelic acid (**35**) was selected to test the capillaries’ performance in terms of repeatability as it was nearly baseline resolved on both columns. Reproducibility of the retention times of both 4-hydroxy-3-methoxymandelic acid (**35**) peaks was satisfactory. In the run-to-run repeatability using one column, the average retention times for the two peaks were 23.5 min (RSD = 1.7%) and 30.6 min (RSD = 1.27%); respectively. In column-to-column repeatability, the average retention times for the two peaks are 23.5 min (RSD = 2.2%) and 30.6 min (RSD = 1.9%); respectively. In batch-to-batch repeatability, the average retention times for peak one and peak two are 22.5 min (RSD = 3.9%) and 31.4 min (RSD = 5.3%); respectively. The retention times and relative standard deviations (RSD) of the retention times ranged between 1.2% and 5.3%. These results suggest that the monolithic capillary columns can be used for reproducible routine analysis. It is worth mentioning that the acceptable %RSD values for intra-batch and inter-batch are 2.5% and 15%; respectively. Furthermore, the column loadability was tested by injecting more than 300 runs on the same column; 4-hydroxy-3-methoxymandelic acid (**35**) was injected in different orders started at run number 160 and ended by run number 307. The same separation was achieved (Figure 5).

### 2.4. Effect of the Concentration of Chiral Selector

The optimum concentration of the colistin sulfate in the polymerization mixture was selected after the preparation of three different capillaries with different concentrations of colistin sulfate (10, 20 and 30 mg/mL). The results revealed that 10 mg/mL afforded better separation and resolution while increasing the concentration to 30 mg/mL or more resulted in poor separation and resolution.

## 3. Discussion

Various macrocyclic antibiotics have been previously synthesized and applied on silica or polymer surfaces as a stationary phase either by immobilization, coating or by covalent bonding [24,28,32,33,34,35,36]. Whilst coating or physical adsorption is considered an suitable method to prepare CSPs, covalent bonding increases the chances for using diverse mobile phases and creates a more robust CSP [46]. It is worth pointing out that most of the CSPs have been prepared via immobilization to bond the chiral selectors to the solid supports. This has resulted in robust and more stable CSP, however, time consuming process offering less coverage of the CS compared to the one pot technique [47]. The way the CSP has been prepared (coating vs. bonding) may affect the chiral recognition mechanism. Thus, bonded-type phase showed a lower chiral recognition power than the coated-type phase.

Schmid et al. have been reporting since 2006 the development of dynamically-coated chiral stationary phases [48] using a macrocyclic antibiotic, namely vancomycin. Few macrocyclic antibiotics were previously used in preparation of chiral monolithic columns for the enantioselective separation of racemic pharmaceuticals [38]. Of interest, in 2010 Schmid et al. [32] published an article describing the preparation of a new chiral stationary phase by dynamic coating of a reversed-phase HPLC monolithic column with vancomycin-derivatives as chiral selector. They then investigated the separation of amino acids using reversed phase chromatographic conditions, namely triethyl-ammonium acetate (TEAA) buffer and methanol. As the underivatized vancomycin is hydrophilic, it could not be adsorbed on the commercial hydrophobic chromolith monolith. Consequently, vancomycin was derivatized to *N*-(2-hydroxydodecyl)-derivative before immobilization on the chromolith. Vancomycin is reversibly adsorbed via a hydrophobic side chain to the reversed-phase material. Similarly, Haroun et al. [49] dynamically coated the macrocyclic antibiotic teicoplanin on RP_18_ and RP_8_ columns. Teicoplanin has a hydrophobic C10 side chain which is attached to the glucopyranosyl group (Figure 1). This side chain was used to immobilize the chiral selector on the hydrophobic reversed phase material. This dynamically coated phase was used for the separation of aromatic amino acids. Similary, in this manuscript, colistine possesses a C9 hydrophobic side chain that can be used for the immobilization on the hydrophobic monolith prepared in this manuscript. It is worth to note that (1) continuous polymers formed from hydrophobic monomers can be used as stationary phase in reversed phase chromatography (RPC) and (2). Solvents used for dissolving the chiral sector can be used as mobile phase (not in excess) on the same column [32].

The chiral recognition of macrocyclic antibiotics used as chiral selectors for the enantio-separation of different compounds is due to the presentation of ionisable acidic or basic functional groups contributing to stereoselectivity, the presence of multiple stereogenic centers, and the presence of both hydrophobic and hydrophilic groups. Therefore, the transient non-covalent diastereomeric complexes with macrocyclic antibiotic are based on both electrostatic interactions and secondary interactions such as hydrophobic, hydrogen bonds, dipole-dipole, π–π interactions, and steric repulsion. Macrocyclic antibiotics have been successfully applied to HPLC and also to CEC for chiral separation of pharmaceutical drugs using stationary phases in the reversed-phase and the normal-phase modes [50,51,52,53,54,55].

Colistin sulfate has never been used as chiral selector although it possesses many points of interaction which significantly increase its enantiorecognition ability. It is well established that under reversed phase conditions, the formation of inclusion complexes within the cavity is the most predominant mechanism of retention and enantioselectivity. Moreover, the presence of different functional groups creates more points of interaction between the enantiomers and the CSP via π–π bonding, hydrogen bonding, dipole–dipole stacking, etc. which can increase the selectivity towards some analytes. For example, in miconazole (**16**), hydrophobic interactions are the prevailing CSP-analyte interactions, whereas hydrogen bonding seems to be more important in the enantiointeractions between the other analytes and CSPs [32]. Initial testing with mixture of methanol-based mobile phase, enantioselective separation was observed for many analytes with polar groups including acebutolol (**4**), indoprofen (**10**), hexaconazole (**15**), normetanephrine (**21**), aminoglutethimide (**22**), propafenone (**26**), tyrosine (**29**), *O*-methoxymandelic acid (**34**) and 4-hydroxy-3-methoxymandelic acid (**35**). This confirms the importance of solvent polarity in the chiral separation mechanism in terms of the inclusion complex stability. The large retention times observed is due to the very low flow rate used. Higher flow rate may result in high backpressure. Peak tailing of the more retained isomers was overcome by the use of mobile phase additives such as triethanol- amine (TEA), which resulted in improved resolution, although, their negative effect on the lifetime of the capillary columns as well as its potential problems with nano-LC systems (e.g. precipitation in the pumps and valves) [56] can be dominant. No remarkably peak tailing of acebutolol (**4**), atenolol (**5**) and tyrosine (**29**) racemates was observed, ascribed to the existence of the amino groups on the surface of the monolithic matrices. It was also observed that the chiral separation was mostly obtained at high water content of the mobile phase; this indicates that water facilitates the interaction between the CSP and the racemates. We postulate that chiral separation in this study was mainly achieved via the formation of inclusion complexes as discussed previously. The use of normal organic phase resulted in high back pressure and very short life time of the prepared column. Nevertheless, the use of *n*-hexane/2-propanol mobile phase mixture ranging from 10–90% (*v*/*v*) resulted in resolution less than 1.

## 4. Experimental

### 4.1. Reagents and Materials

Colistin sulfate (99%), ethylene glycol dimethacrylate (EGDMA, 98%), glycidyl methacrylate (GMA, 98%), 1-propanol (99%), 1,4-butanediol (99%), trifluoroacetic acid (TFA, ≥99.5%), sodium hydroxide and hydrochloric acid were purchased from Sigma Aldrich (Milwaukee, WI, USA). Acetone (AR grade) and ethanol (HPLC grade) were purchased from BDH (Kilsyth, Vic., Australia). Methanol (HPLC) grade was purchased from Scharlau (Sentmenat, Spain). All other reagents were of the highest available grade and used as received. The fused-silica capillaries (150 µm internal diameter) were purchased from Polymicro Technologies (Phoenix, AZ, USA). 2,2-Azobis(isobutyronitrile) (AIBN) was obtained from Wako (Osaka, Japan). Water used for dilutions and experiments was purified by a Nano-pure Infinity water system (NJ, USA). The racemic analytes were mostly purchased from Sigma Aldrich.

### 4.2. Preparation and Characterization of the Monolithic Columns

#### 4.2.1. Activation of the Fused Silica Capillaries

Briefly, the fused silica capillaries were rinsed using a Harvard syringe pump (Harvard Apparatus, Holliston, MA, USA) and a 250 µL gas-tight syringe (Hamilton Company, Reno, NE, USA) with acetone and water 2–3 times each, activated with 0.2 mol/L sodium hydroxide (NaOH) for 6 h confirming the absence of any air bubbles, washed with water 3–4 times till neutral (pH 7), then washed with 0.2 mol/L hydrochloride (HCl) for 12 h, rinsed with water and ethanol 2–3 times each. A 20% (*w*/*w*) solution of 3-(trimethoxysilyl)propyl methacrylate in 95% ethanol adjusted to pH 5 using acetic acid was pumped through the capillaries at a flow rate of 0.25 µL/min for 6 h. The capillary was then washed with acetone one time and dried with a stream of nitrogen for 2 min. then left at room temperature for 24 h.

#### 4.2.2. Preparation of Colistin Sulfate Functionalized Monomer

**Figure 6:**
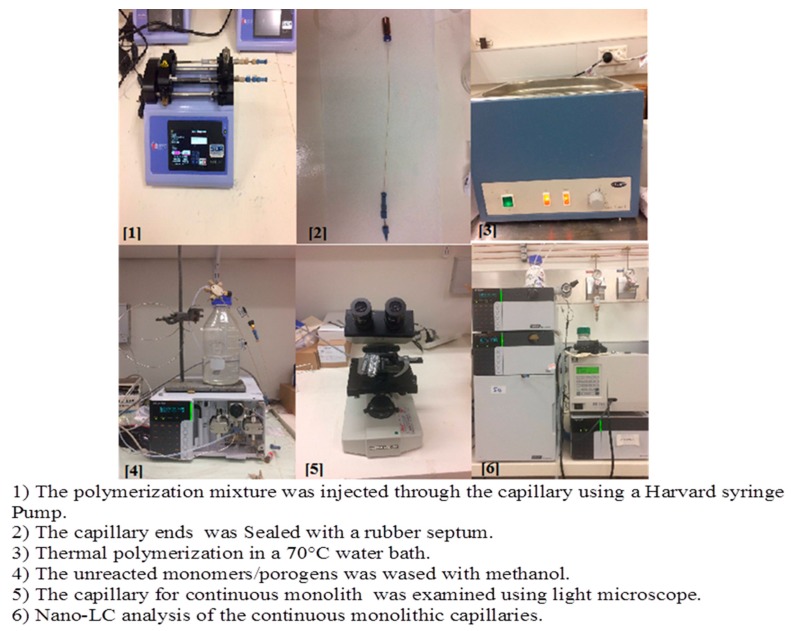
Steps showing the preparation of polymer monolithic capillary columns.

The short (∼25 cm in length) surface modified capillary was filled by Harvard syringe pump with the degassed polymerization mixture at 0.25 µL/min using the syringe pump. Colistin sulfate polymer-based monolithic capillary column was prepared via in situ copolymerization of binary monomer mixtures consisted of GMA (20%) as a monomer and EGDMA (20%) as across linker along with different porogens namely; 1-propanol (48%), 1,4-butanediol (6%), in the presence of 1 wt% AIBN (with respect to monomers) and colistin sulfate (6%) as chiral selector. The blank column (G column) was prepared using the same procedure by addition of water (6%) instead of water. The filled capillaries were then sealed with a septum, placed in 70 °C water bath for 18 h for the polymerization reaction to take place. The unreacted monomers were removed from the monolithic columns by pumping methanol at a flow rate of 100 µL/h for 24 h before being investigated under light microscope to ensure its consistency and homogeneity of the polymerization mixture inside the capillary. The filled capillaries were conditioned with mobile phase for 1–3 days at µL/min (Figure 6). The ratios of the monomers to the porogens were kept 40% and 60%, respectively. The ratios of the porogens were fixed as 48% 1-propanol, 6% 1,4-butanediol and 6% chiral selector, all percentages are *w*/*w*.

#### 4.2.3. SEM of the Prepared Monoliths

SEM was performed to study the morphology of the prepared capillaries. The capillaries were cut into ~1 cm sections and put perpendicularly on 12.7 mm pin-type aluminum stub using double face epoxy resin tape. SEM was carried out and high resolution images were collected by sputter coating the capillary sections with gold Using ZEISS SIGMA FE-SEMs for High Quality Imaging and Advanced Analytical Microscopy (ZEISS Sigma, Jena, Germany).

### 4.3. Instrumentation

A nano-liquid chromatographic system consisting of an LC-10AD VP pump (Shimadzu, Kyoto, Japan), injector model 7725i-049 (Rheodyne, Park Court, CA, USA), a UV-Vis detector model MU 701 UV-VIS (GL Science, Tokyo, Japan) and a Shimadzu CDM-20A communications bus module was used. The system flow was split after direct injection. The data was processed by the Shimadzu Lab-Solutions software version 5.54 SP2 (Shimadzu, Kyoto, Japan).

### 4.4. Standard Solutions and Sample Preparation

Stock solutions of the racemic analytes at concentrations of 1 mg/mL in filtered HPLC grade methanol were prepared. Prior to injection, the stock solutions were further diluted 10× by mobile phase and filtered through Minisart RC 15 0.2 µm pore size filters (Sartorius, Goettingen, Germany). Tested compounds: β-blockers: alprenolol (**1**), metoprolol (**2**), propranolol (**3**), acebutolol (**4**), atenolol (**5**); α-blockers: naftopidil (**6**); anti-inflammatory drugs: ibuprofen (**7**), naproxen (**8**), flurbiprofen (**9**), indoprofen (**10**), cizolirtine (**11**), cizolirtine citrate (**12**), carprofen (**13**), glafenine (**14**); antifungal drugs: hexaconazole (**15**), miconazole (**16**), diniconazole (**17**) sulconazole (**18**); norepinephrine-dopamine reuptake inhibitor: nomifensine (**19**); catecholamines: arterenol (**20**), normetanephrine (**21**); sedative hypnotics: aminoglutethimide (**22**); anti-histamines: chlorpheneramine (**23**); anticancer drugs: ifosfamide (**24**); antiarrhythmic drugs: tocainide (**25**), propafenone (**26**); flavonoids: flavanone (**27**); amino acids: glutamic acid monohydrate (**28**), tyrosine (**29**), phenylalanine (**30**); anti-platelet agents: clopidogrel (**31**); immunomodulatory drugs: thalidomide (**32**); miscellaneous: 1-acenaphthenol (**33**), *O*-methoxymandelic acid (**34**) 4-hydroxy-3-methoxymandelic acid (**35**), 1-indanol (**36**) and ampicillin (**37**). The chemical structures of the investigated racemates are shown in Figure 3.

### 4.5. HPLC Conditions

The mobile phase consisted of water/methanol (*v*/*v*) for the reversed phase HPLC and *n*-hexane/2-propanol for normal phase HPLC. For all samples, the injected volume was 0.2 µL at room temperature with flow rate 1 μL/min on C1 capillary column (150 µm ID, 25 cm length). Preliminary UV analyses were performed at a wavelength of 219 nm.

## 5. Conclusions

The macrocyclic antibiotic colistin sulphate has been used for the first time as a chiral selector entrapped in a polymer monolith for enantioselective capillary chromatography. The new capillary column was investigated for the enantioselective separation of a set of racemic drugs. Acceptable separation was achieved for many drugs, including acebutolol (**4**), ibuprofen (**7**), indoprofen (**10**), hexaconazole (**15**), miconazole (**16**), normetanephrine (**21**), aminoglutethimide (**22**), propafenone (**26**), tyrosine (**29**), *O*-methoxymandelic acid (**34**) and 4-hydroxy-3-methoxymandelic acid (**35**) under reversed phase chromatographic conditions, while normal phase conditions did not achieve any acceptable separations. The method provides more economical analysis under environmentally benign reversed phase conditions.

## Figures and Tables

**Table 1:** Chromatographic data, separation and resolution factors for the significantly resolved compounds, using reversed mobile phases, flow rate: 1 µL/min.

Column C1 (Colistin Sulfate)						
Phase	Mobile Phase	Drug	Rt_1_ (min)	Rt_2_ (min)	Separation Factor (α)	Resolution (Rs)
**Reversed Phase**	Methanol:water 80:20	Ibuprofen (**7**)	23.3	38.9	1.7	1.02
	Methanol:water 40:60	Indoprofen (**10**)	24.3	40.1	1.72	1.6
		Hexaconazole (**15**)	23.8	37.6	1.9	1.2
		Miconazole (**16**)	17.5	23.6	1.4	1.6
	Methanol:water 30:70	Indoprofen (**10**)	23.6	63.4	2.8	1.63
		Miconazole (**16**)	17.6	23.6	1.41	1.6
	Methanol:water 10:90	Aminoglutethimide (**22**)	22.4	33.6	1.5	1
		Tyrosine (**29**)	23.4	35	1.5	1.64
		*O*-Methoxymandelic acid (**34**)	26.1	40.2	1.52	1.1
	Methanol:water 10:90, 1%TEA	Acebutolol (**4**)	22.8	28.7	1.3	1.14
		Normetanephrine (**21**)	22.3	28.4	1.3	1.3
		Propafenone (**26**)	21.4	30.5	1.3	1.2
		Tyrosine (**29**)	18.1	24.2	1.3	1.7
		4-Hydroxy-3-methoxymandelic acid (**35**)	24.1	31.5	1.3	1.03

## References

[1] Lin G.Q., Zhang J.G., Cheng J.F. (2011). Overview of chirality and chiral drugs. Chiral Drugs Chem. Biol. Action.

[2] Maier N.M., Franco P., Lindner W. (2001). Separation of enantiomers: Needs, challenges, perspectives. J. Chromatogr. A.

[3] Lorenz H., Seidel-Morgenstern A. (2014). Processes to separate enantiomers. Angew. Chem. Int. Ed..

[4] Ali I., Aboul-Enein H.Y. (2005). Chiral Pollutants: Distribution, Toxicity and Analysis by Chromatography and Capillary Electrophoresis.

[5] Aboul-Enein H., Wainer I. (1997). The Impact of Stereochemistry on Drug Development and Use.

[6] Francotte E.R. (2001). Enantioselective chromatography as a powerful alternative for the preparation of drug enantiomers. J. Chromatogr. A.

[7] Gil-Av E., Feibush B. (1967). Resolution of enantiomers by gas liquid chromatography with optically active stationary phases. Separation on packed columns. Tetrahedron Lett..

[8] Ward T.J., Ward K.D. (2011). Chiral separations: A review of current topics and trends. Anal. Chem..

[9] Speybrouck D., Lipka E. (2016). Preparative supercritical fluid chromatography: A powerful tool for chiral separations. J. Chromatogr. A.

[10] Fanali S. (2017). Nano-liquid chromatography applied to enantiomers separation. J. Chromatogr. A.

[11] Lämmerhofer M. (2010). Chiral recognition by enantioselective liquid chromatography: mechanisms and modern chiral stationary phases. J. Chromatogr. A.

[12] Catani M., Ismail O.H., Gasparrini F., Antonelli M., Pasti L., Marchetti N., Felletti S., Cavazzini A. (2017). Recent advancements and future directions of superficially porous chiral stationary phases for ultrafast high-performance enantioseparations. Analyst.

[13] Peluso P., Mamane V., Aubert E., Cossu S. (2017). Recent trends and applications in liquid phase chromatography enantioseparation of atropisomers. Electrophoresis.

[14] Glajch J.L., Kirkland J.J., Köhler J. (1987). Effect of column degradation on the reversed-phase high-performance liquid chromatographic separation of peptides and proteins. J. Chromatogr. A.

[15] Wehrli A., Hildenbrand J.C., Keller H.P., Stampfli R., Frei R.W. (1978). Influence of organic bases on the stability and separation properties of reversed-phase chemically bonded silica gels. J. Chromatogr. A.

[16] McNeff C., Zigan L., Johnson K., Carr P.W., Wang A., Weber-Main A.M. (2000). Analytical advantages of highly stable stationary phases for reversed-phase LC. LC GC.

[17] Cabrera K. (2004). Applications of silica-based monolithic HPLC columns. J. Sep. Sci..

[18] Núñez O., Nakanishi K., Tanaka N. (2008). Preparation of monolithic silica columns for high-performance liquid chromatography. J. Chromatogr. A.

[19] Kanatyeva A.Y., Kurganov A.A., Viktorova E.N., Korolev A.A. (2008). Monolithic stationary phases in liquid and gas chromatography. Russ. Chem. Rev..

[20] Wistuba D. (2010). Chiral silica-based monoliths in chromatography and capillary electrochromatography. J. Chromatogr. A.

[21] Nakanishi K., Soga N. (1992). Phase separation in silica sol-gel system containing polyacrylic acid I. Gel formaation behavior and effect of solvent composition. J. Non-Cryst. Solids.

[22] Turson M., Zhou M., Jiang P., Dong X. (2011). Monolithic poly(ethylhexyl methacrylate-co-ethylene dimethacrylate) column with restricted access layers prepared via reversible addition-fragmentation chain transfer polymerization. J. Sep. Sci..

[23] Guiochon G. (2007). Monolithic columns in high-performance liquid chromatography. J. Chromatogr. A.

[24] Gibson G.T.T., Mugo S.M., Oleschuk R.D. (2008). Surface-mediated effects on porous polymer monolith formation within capillaries. Polymer.

[25] Saunders K.C., Ghanem A., Hon W.B., Hilder E.F., Haddad P.R. (2009). Separation and sample pre-treatment in bioanalysis using monolithic phases: A review. Anal. Chim. Acta.

[26] Liao J.-L., Zhang R., Hjertén S. (1991). Continuous beds for standard and micro high-performance liquid chromatography. J. Chromatogr. A.

[27] Peters E.C., Petro M., Svec F., Fréchet J.M. (1997). Molded Rigid Polymer Monoliths as Separation Media for Capillary Electrochromatography. Anal. Chem..

[28] Svec F. (2010). Porous polymer monoliths: amazingly wide variety of techniques enabling their preparation. J. Chromatogr. A.

[29] Fouad A., Marzouk A.A., Ibrahim S.M., Sobhy M., Ghanem A. (2017). Functionalized polymer monoliths with carbamylated amylose for the enantioselective reversed phase nano-liquid chromatographic separation of a set of racemic pharmaceuticals. J. Chromatogr. A.

[30] Ahmed M., Ghanem A. (2014). Chiral β-cyclodextrin functionalized polymer monolith for the direct enantioselective reversed phase nano liquid chromatographic separation of racemic pharmaceuticals. J. Chromatogr. A.

[31] Ahmed M., Yajadda M.M.A., Han Z.J., Su D., Wang G., Ostrikov K.K., Ghanem A. (2014). Single-walled carbon nanotube-based polymer monoliths for the enantioselective nano-liquid chromatographic separation of racemic pharmaceuticals. J. Chromatogr. A.

[32] Pittler E., Schmid M.G. (2010). Enantioseparation of dansyl amino acids by HPLC on a monolithic column dynamically coated with a vancomycin derivative. Biomed. Chromatogr..

[33] Armstrong D.W., Nair U.B. (1997). Capillary electrophoretic enantioseparations using macrocyclic antibiotics as chiral selectors. Electrophoresis.

[34] Fedorova I.A., Shapovalova E.N., Shpigun O.A., Staroverov S.M. (2016). Bovine serum albumin adsorbed on eremomycin and grafted on silica as new mixed-binary chiral sorbent for improved enantioseparation of drugs. J. Food Drug Anal..

[35] Ilisz I., Grecsó N., Forró E., Fülöp F., Armstrong D.W., Péter A. (2015). High-performance liquid chromatographic separation of paclitaxel intermediate phenylisoserine derivatives on macrocyclic glycopeptide and cyclofructan-based chiral stationary phases. J. Pharm. Biomed. Anal..

[36] Hroboňová K., Lehotay J., Čižmárik J. (2016). HPLC Enantioseparation of Phenylcarbamic Acid Derivatives by Using Macrocyclic Chiral Stationary Phases. Nova Biotechnol. et Chim..

[37] Fedorova I., Shapovalova E., Shpigun O. (2017). Separation of β-blocker and amino acid enantiomers on a mixed chiral sorbent modified with macrocyclic antibiotics eremomycin and vancomycin. J. Anal. Chem..

[38] Hsieh M.L., Chau L.K., Hon Y.S. (2014). Single-step approach for fabrication of vancomycin-bonded silica monolith as chiral stationary phase. J. Chromatogr. A.

[39] Qin F., Xie C., Yu Z., Kong L., Ye M., Zou H. (2006). Monolithic enantiomer-selective stationary phases for capillary electrochromatography. J. Sep. Sci..

[40] Claude Guillaume Y., André C. (2013). Fast enantioseparation by HPLC on a modified carbon nanotube monolithic stationary phase with a pyrenyl aminoglycoside derivative. Talanta.

[41] Ghanem A., Adly F.G., Sokerik Y., Antwi N.Y., Shenashen M.A., El-Safty S.A. (2017). Trimethyl-β-cyclodextrin-encapsulated monolithic capillary columns: Preparation, characterization and chiral nano-LC application. Talanta.

[42] Hroboňová K., Deáková Z., Moravčík J., Lehotay J., Armstrong D.W., Lomenová A. (2015). Separation Of Methionine Enantiomers By Using Teicoplanin And Cyclofructan Columns. Nova Biotechnol. et Chim..

[43] Maia A.S., Castro P.M., Tiritan M.E. (2016). Integrated liquid chromatography method in enantioselective studies: Biodegradation of ofloxacin by an activated sludge consortium. J. Chromatogr. B.

[44] Fanali C., Fanali S., Chankvetadze B. (2016). HPLC Separation of Enantiomers of Some Flavanone Derivatives Using Polysaccharide-Based Chiral Selectors Covalently Immobilized on Silica. Chromatographia.

[45] Harvanová M., Gondová T. (2017). New enantioselective LC method development and validation for the assay of modafinil. J. Pharm. Biomed. Anal..

[46] Chankvetadze B., Kubota T., Ikai T., Yamamoto C., Kamigaito M., Tanaka N., Nakanishi K., Okamoto Y. (2006). High-performance liquid chromatographic enantioseparations on capillary columns containing crosslinked polysaccharide phenylcarbamate derivatives attached to monolithic silica. J. Sep. Sci..

[47] Zhang Z., Wu M., Wu R.A., Dong J., Ou J., Zou H. (2011). Preparation of perphenylcarbamoylated β-cyclodextrin-silica hybrid monolithic column with “one-pot” approach for enantioseparation by capillary liquid chromatography. Anal. Chem..

[48] Schmid M.G., Schreiner K., Reisinger D., Gübitz G. (2006). Fast chiral separation by ligand-exchange HPLC using a dynamically coated monolithic column. J. Sep. Sci..

[49] Haroun M., Ravelet C., Grosset C., Ravel A., Villet A., Peyrin E. (2006). Reversal of the enantiomeric elution order of some aromatic amino acids using reversed-phase chromatographic supports coated with the teicoplanin chiral selector. Talanta.

[50] Dermaux A., Lynen F., Sandra P. (1998). Chiral capillary electrochromatography on a vancomycin stationary phase. J. Sep. Sci..

[51] Karlsson C., Karlsson L., Armstrong D.W., Owens P.K. (2000). Evaluation of a vancomycin chiral stationary phase in capillary electrochromatography using polar organic and reversed-phase modes. Anal. Chem..

[52] Desiderio C., Aturki Z., Fanali S. (2001). Use of vancomycin silica stationary phase in packed capillary electrochromatography I. Enantiomer separation of basic compounds. Electrophoresis.

[53] Fanali S., Catarcini P., Quaglia M.G. (2002). Use of vancomycin silica stationary phase in packed capillary electrochromatography: III. Enantiomeric separation of basic compounds with the polar organic mobile phase. Electrophoresis.

[54] Karlsson C., Wikström H., Armstrong D.W., Owens P.K. (2000). Enantioselective reversed-phase and non-aqueous capillary electrochromatography using a teicoplanin chiral stationary phase. J. Chromatogr. A.

[55] Wikström H., Svensson L.A., Torstensson A., Owens P.K. (2000). Immobilisation and evaluation of a vancomycin chiral stationary phase for capillary electrochromatography. J. Chromatogr. A.

[56] Si Ahmed K., Tazerouti F., Badjah-Hadj-Ahmed A.Y., Meklati B.Y. (2007). Preparation and chromatographic properties of a multimodal chiral stationary phase based on phenyl-carbamate-propyl-β-CD for HPLC. J. Sep. Sci..

